# Pediatric alternating allergic fungal rhinosinusitis: A case report and literature review

**DOI:** 10.1016/j.ijscr.2018.11.015

**Published:** 2018-11-16

**Authors:** Danah H. Althomaly, Ali A. AlMomen

**Affiliations:** a Medical intern, Imam Abdulrahman Bin Faisal University, Khobar, Saudi Arabia; b Consultant ENT, Rhinology and Endoscopic Skull Base Surgery at King Fahad Specialist Hospital, Dammam, Saudi Arabia

**Keywords:** AFRS, allergic fungal rhinosinusitis, KFSHD, King Fahad Specialist Hospital Dammam, AFS, allergic fungal sinusitis, Alternating, Contralateral, Allergic fungal sinusitis, Fungal infection, Pediatric, Case report

## Abstract

**Objectives:**

to report the alternating nature of allergic fungal rhinosinusitis in children in the Eastern part of Saudi Arabia and to review the experience of King Fahad Specialist Hospital in the diagnosis and management of alternating allergic fungal rhinosinusitis in children.

An 8 years old Saudi girl with alternating allergic fungal rhinosinusitis was diagnosed and managed. The patient was diagnosed to have unilateral left allergic fungal rhinosinusitis and underwent endoscopic sinus surgery and cleaning of the left sinuses from polyps, mud and mucin. One year postoperatively the patient developed AFRS in the contralateral right side.

**Conclusion:**

involvement of the contralateral sinuses in children with AFRS is uncommon. The normal uninvolved sinus should be involved in the routine endoscopic examination and the post-operative treatment in order to minimize the risk of disease recurrence.

## Highlights

• Allergic fungal rhinosinusitis nature in children is more aggressive when compared to adults.
• Endoscopic sinus surgery is an important therapeutic step in the treatment of allergic fungal rhinosinusits.
• The reason for this contralateral development of AFRS not clear, but it may be part of the natural disease process.
• Involvement of the contralateral sinuses in children is uncommon. The normal uninvolved sinus should be involved in the routine endoscopic examination and the post-operative treatment in order to minimize the risk of disease recurrence.

## 1 Introduction

Allergic fungal rhinosinusitis (AFRS) was first reported as a distinct clinical entity in 1976 [1]. It is coupled with the clinical entity of fungus ball (mycetoma) as a form of noninvasive fungal sinus disease, separate from and unrelated to invasive fungal sinus pathology. AFRS is a truly unique pathologic entity, defined largely by the presence of allergic fungal mucin, which is a thick, tenacious, eosinophilic secretion with characteristic histologic findings. AFRS develops in young adults and adolescents primarily, being more common in temperate regions with high humidity. Initially, the primary causative organism was thought to be Aspergillus [2]. There are several studies in the literature regarding AFRS in adults. However even though it presents as well but to lesser extent in the pediatric age group, there only a few data in literature regarding its nature, clinical course and recurrence in children [3]. This case report focus on uncommon clinical presentation of AFRS in pediatric age group. This case was diagnosed and managed at King Fahad Specialist Hospital (KFSHD), a tertiary care hospital in Al-Dammam, Saudi Arabia.

## 2 Case report

**Fig. 1:**
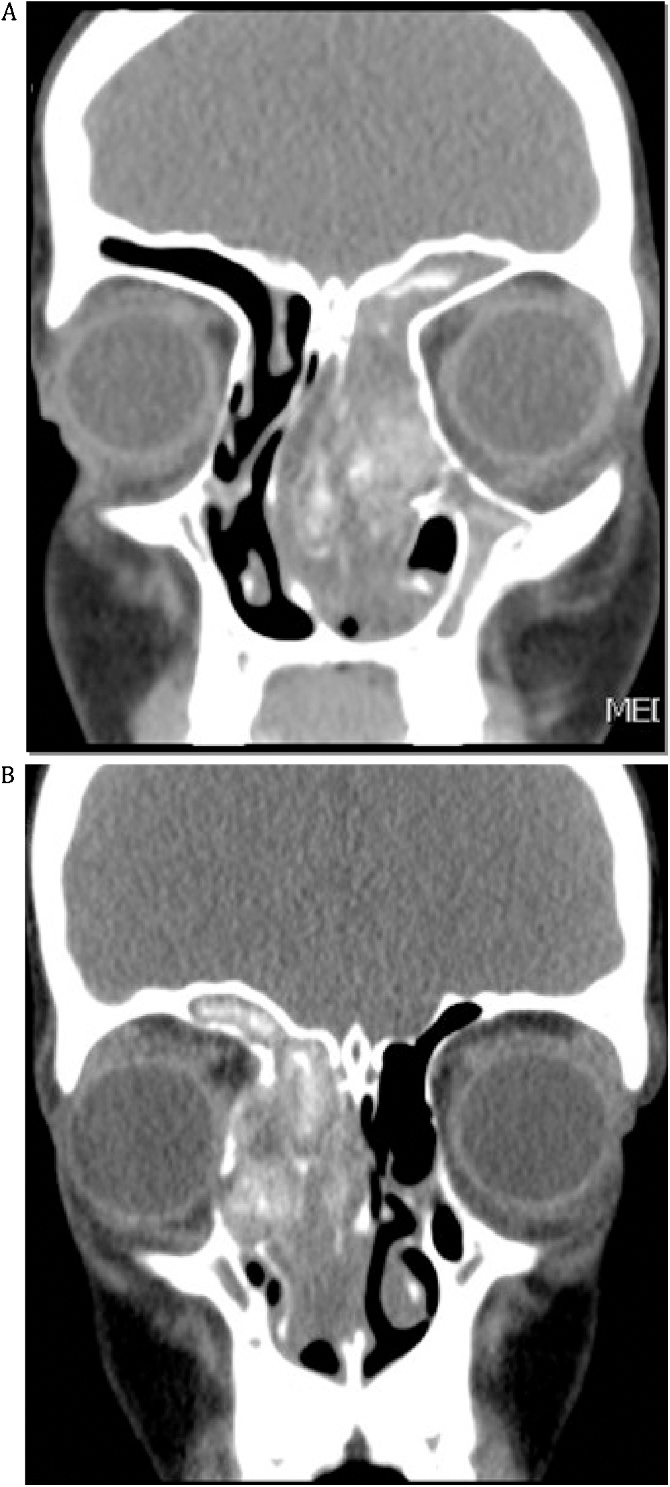
(**A)** Coronal CT image shows heterogeneous opacity filling the left maxillary and frontoethmoidal sinuses with bone expansion and pushing the septum to the right side. (**B)** Coronal CT image post-surgery shows disease recurrence in the right sinuses and clean left side.

8 years old Saudi girl known case of bronchial asthma, complaining of left nasal obstruction with mild eye proptosis for 9 months noticed by her parents. Her past medical and surgical histories were unremarkable. Drug history, family history and psychological history were insignificant. Examination showed left eye proptosis, enlarged medial canthus and polyps filling the left nasal cavity. CT sinuses showed a heterogeneous opacity of the left maxillary and frontoethmoidal sinuses with bone expansion and obliteration of the left nasal cavity (Fig. 1A) consistent with AFRS.

The patient and her family were fully counseled about the nature of the disease, the surgical management, treatment plans and recurrence. The patient underwent endoscopic sinus surgery and cleaning of the left sinuses from polyps, mud and mucin, performed by A.A. (rhinologist). Culture was positive for asperigillus. Initially the patient was doing well for one year follow up. However, one year later she started to complain of right nasal discharge and obstruction. Examination showed clear left nasal cavity with no recurrence of disease but there were right nasal polyps with mucin. CT sinuses showed recurrence of the disease in the right side with clear left nasal cavity (Fig. 1B).

The patient underwent endoscopic sinus surgery for right sinuses cleaning and polyp’s removal. Culture was positive for asperigillus. The patient had no recurrence after 3 years follow up.

## 3 Discussion

Allergic fungal sinusitis (AFS) is a benign, extramucosal, non-invasive fungal sinus disease representing an allergic/hypersensitivity response to extramucosal fungal pathogens in the sinus [4]. As for the presentation of AFS in children, it may be quite subtle with the onset typically being an indolent process. Children report a slow onset of nasal airway obstruction and production of large, dark-colored nasal debris [4,5]. However, Patro, et al. presented a prospective study of 50 patients divided into pediatric group (6-14yrs) and adult group (15-65yrs) to compare between the aggressiveness of the disease. It showed that pediatric group had a less mean duration of symptoms, telecanthus and proptosis was more common, and Lund-MacKay radiological scoring was higher. Based on that, they concluded the more aggressive course in pediatric age group when compared to adults with involvement of multiple sinuses [6]. In another prospective study of 200 cases divided into 2 groups. 68 cases in group 1 (less than 15 years) and 132 cases in group 2 (more than 15 years). The study concluded the following; nasal obstruction was the most common presentation in both groups. The children had higher incidence of having unilateral disease compared with adults, and finally group 1 had higher incidence facial deformities, proptosis, and intraorbital/intracranial extension along with higher rate of recurrence which also suggested a more aggressive nature of AFRS in children when compared to adults as the previous study [5]. Several other studies in the literature has reported alteration in the facial skeleton in 42% of pediatric patients which includes Proptosis, telecanthus, or malar flattening, compared to 10% for adults. This could be explained by the more pliable bony structure of children [4,7,8]. Treatment of AFS in pediatric cases does not differ from that in adults, and endoscopic sinus surgery is an important therapeutic step. Surgical debulking of diseased tissue, removal of fungal debris from involved sinuses, and establishment of normal mucociliary drainage pathways are the main goals [9]. In the post-operative period, saline nasal irrigation, nasal corticosteroid sprays, and oral steroids are administrated to avoid recurrence [7]. Antifungal nasal agents such as fluconazole seem to be useful in preventing progression of disease and/or improving symptoms, but to date limited data is available [7]. In addition to medical treatments, endoscopic cleanings should be periodically scheduled after surgery, and patients should be followed for a very long period [10]. Recurrence of allergic fungal rhinosinusitis (AFRS) is well recognized. However, there is scarcity in the literature describing involvement of the non-diseased sinuses. AlQahtani A. et al. presented a case-control study of 68 patients. It showed that delayed contralateral involvement after the initial surgery was found in 30.8% with mean duration of recurrence 16.9 months [11]. A significant association was found with the presence of pre-operative contralateral symptoms and signs of inflammation. Post-operative use of budesonide irrigation was associated with less contralateral involvement. Association of other variables like; comorbidities, perioperative use of systemic steroid, radiological signs, extent of surgery, additional surgery to the contralateral side, and post-operative use of systemic steroids did not show statistical significance. Involvement of the contralateral sinuses in 30% of unilateral AFRS cases is considered significant. The non-diseased sinuses should be involved in the routine endoscopic examination and post-operative treatment. In other retrospective analysis conducted by Marglani O. et al. AFRS was diagnosed in 52 (38.2%) out of 136 cases of chronic rhinosinusitis with or without nasal polyps treated with endoscopic sinus surgery. Out of the 52 AFRS patients, 16 (30.8%) cases presented with unilateral AFRS, and all were treated with standard surgical and medical therapy. During a mean follow-up of 24.8 months, nine (56.2%) of the 16 unilateral cases remained disease-free, four (25%) developed AFRS on the contralateral side, two (12.5%) had recurrent ipsilateral AFRS, and one (6.25%) had both ipsilateral recurrence and contralateral development of AFRS [12].

The reason for this contralateral development of AFRS is not clear, but it may be part of the natural disease process. We postulate that unilateral AFRS is an early stage in disease presentation that can eventually progress to bilateral disease. Contralateral development of AFRS may also be theoretically explained as the transfer of fungal antigens from the affected side to the healthy side because of intraoperative or postoperative irrigations. However, this phenomenon should alert the surgeon that future development of contralateral AFRS needs to be part of patients’ preoperative counseling. In addition, postoperative follow-up should be directed to both sinonasal passages. A challenging question arises whether providing a prophylactic treatment to the contralateral side may have a role in certain cases such as aggressive disease or questionable follow-up. Clearly, current study findings do not justify operating normal uninvolved sides [12].

## 4 Conclusion

To conclude involvement of the contralateral sinuses in children is uncommon. The normal uninvolved sinus should be involved in the routine endoscopic examination and the post-operative treatment in order to minimize the risk of disease recurrence. The reason for this contralateral development of AFRS is not clear, but it may be part of the natural disease process. We assume that unilateral AFRS is an early stage in disease presentation that can eventually progress to bilateral disease.

## Conflict of interest

The authors declare that there is no conflict of interest regarding the publication of this paper.

## Sources of funding

There is no financial support and sponsorship.

## Ethical approval

According to our institution guideline, case report doesn’t require ethical approval.

## Consent

Written informed consent was obtained from the parents for publication of this case report on behalf of the patient.

## Author contribution

Danah H Althomaly: conceptualization, validation, data curation, writing- original draft.

Writing- review and editing, visualization, funding acquisition.

Ali A Almomen: Writing- review and editing, supervision, project administration.

## Registration of research studies

None.

Because this is a case reports not study.

## Guarantor

Dr. Ali A Almomen.

Consultant ENT, Rhinology and Endoscopic Skull Base Surgery At King Fahad Specialist Hospital, Dammam, KSA.

## Data availability

The data used to support the findings of this study are included within the article. Also, they are available from the corresponding author upon request.

## Methods

This work has been reported in line with the SCARE criteria.

## Provenance and peer review

Not commissioned, externally peer reviewed.
